# A Biomimetic, Bilayered Antimicrobial Collagen-Based Scaffold for Enhanced Healing of Complex Wound Conditions

**DOI:** 10.1021/acsami.2c18837

**Published:** 2023-03-31

**Authors:** Matthew McGrath, Karolina Zimkowska, Katelyn J. Genoud, Jack Maughan, Javier Gutierrez Gonzalez, Shane Browne, Fergal J. O’Brien

**Affiliations:** † Tissue Engineering Research Group, Department of Anatomy & Regenerative Medicine, Royal College of Surgeons in Ireland (RCSI), 123 St. Stephen’s Green, Dublin D02 YN77, Ireland; ‡ Advanced Materials and BioEngineering Research (AMBER) Centre, RCSI and TCD, Dublin D02 PN40, Ireland; § Regenerative Medicine Institute, University of Galway, Galway H91 TK33, Ireland; ∥ Trinity Centre for Biomedical Engineering, Trinity College Dublin, Dublin 2 D02 PN40, Ireland; ⊥ School of Physics, Trinity College Dublin, Dublin D02 PN40, Ireland; ¶ Centre for Research on Adaptive Nanostructures and Nanodevices (CRANN), Trinity College Dublin, Dublin 2 D02 W085, Ireland; □ School of Chemistry, University of Dublin, Trinity College Dublin, Dublin 2 D02 W085, Ireland

**Keywords:** wound healing, tissue engineering, collagen, scaffold, chitosan, antimicrobial, angiogenesis

## Abstract

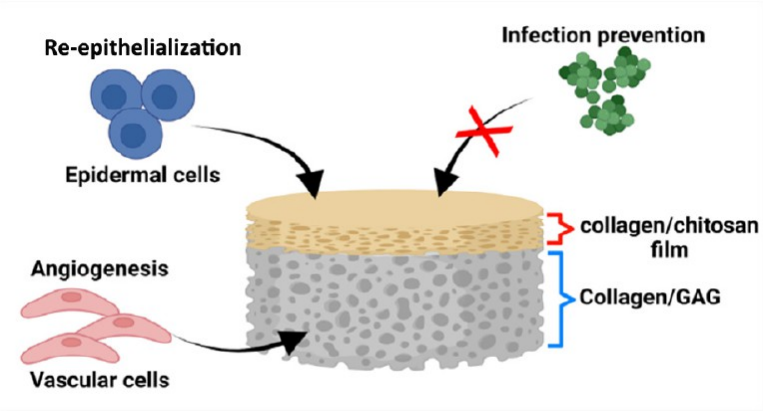

Chronic, nonhealing wounds in the form of diabetic foot ulcers (DFUs) are a major complication for diabetic patients. The inability of a DFU to heal appropriately leads to an open wound with a high risk of infection. Current standards of care fail to fully address either the underlying defective wound repair mechanism or the risk of microbial infection. Thus, it is clear that novel approaches are needed. One such approach is the use of multifunctional biomaterials as platforms to direct and promote wound healing. In this study, a biomimetic, bilayered antimicrobial collagen-based scaffold was developed to deal with the etiology of DFUs. An epidermal, antimicrobial collagen/chitosan film for the prevention of wound infection was combined with a dermal collagen–glycosaminoglycan scaffold, which serves to support angiogenesis in the wound environment and ultimately accelerate wound healing. Biophysical and biological characterization identified an 1-ethyl-3-(3-(dimethylamino)propyl)carbodiimide cross-linked bilayered scaffold to have the highest structural stability with similar mechanical properties to products on the market, exhibiting a similar structure to native skin, successfully inhibiting the growth and infiltration of *Staphylococcus aureus* and supporting the proliferation of epidermal cells on its surface. This bilayered scaffold also demonstrated the ability to support the proliferation of key cell types involved in vascularization, namely, induced pluripotent stem cell derived endothelial cells and supporting stromal cells, with early signs of organization of these cells into vascular structures, showing great promise for the promotion of angiogenesis. Taken together, the results indicate that the bilayered scaffold is an excellent candidate for enhancement of diabetic wound healing by preventing wound infection and supporting angiogenesis.

## 1 Introduction

Diabetes mellitus is a chronic disease characterized by elevated blood glucose levels (hyperglycemia) due to defects in insulin secretion or function.^1^ According to the World Health Organization and the International Diabetes Association, the number of people with diabetes mellitus rose from 108 million in 1980^2^ to 537 million in 2021,^3^ with a projected increase to 700 million adults by 2045.^3^ Diabetic foot ulcers (DFUs) are among the most debilitating complications associated with diabetes.^4^ Loss of sensation in the foot, along with excessive plantar pressure from limited joint mobility and foot deformities, can lead to the development of DFUs,^5^ which occur in 15% of all diabetic patients.^6^ It is estimated that 61% of DFUs become infected^7^ and 15% of those with DFUs require amputation.^8^

Classical wound healing is a dynamic and complex process that follows four main overlapping phases: homeostasis, inflammation, proliferation, and remodeling—ultimately leading to wound closure and restoration of the skin’s barrier function. Each of these phases is essential for successful healing, and chronic, nonhealing wounds arise when a wound fails to progress through the normal steps of wound healing. While dysfunction occurs in many of these phases, insufficient vascularization is one of the key factors that inhibits wound healing in a DFU. Elevated blood glucose levels inhibit normal endothelial cell functionality, leading to micro and macrovascular complications that ultimately impair angiogenesis and wound healing.^9^ Without successful angiogenesis and a stable vasculature within the wound environment, a lack of oxygen and nutrient supply hinders cell proliferation, the formation of a mature extracellular matrix (ECM), and the wound healing process. A lack of healing leaves these open wounds susceptible to infection,^10^ which can cause severe injury to the limb and can ultimately necessitate amputation.

Current standards of general wound care include debridement of the wound bed, optimization of glycaemic control, pressure offloading in the form of total contact casts (most common in DFUs), antibiotic treatment of infection, and maintenance of a moist wound environment.^11^ Despite the array of treatment options available, about 20% of patients have unhealed DFUs after 1 year,^12^ so an alternative approach to treatment is needed. One such alternative is the use of biomaterial scaffolds, wherein the biomaterials act as a template for cell infiltration and tissue regeneration by providing an environment to support the growth of new tissue. Collagen, as the main protein of the ECM and accounting for about three-quarters of the dry weight of skin,^13−15^ is widely used in tissue engineering applications.^16−21^ Through lyophilization,^22,23^ biomimetic collagen–glycosaminoglycan (CG) scaffolds have been produced which promote tissue regeneration through cellular infiltration and neotissue formation. These scaffolds make use of collagen’s biodegradability, weak antigenicity, and cells’ inherent ability to recognize, interact with, and proliferate within collagen-based biomaterials.^24^ This concept has been applied clinically for wound healing, for example, the Integra Dermal Regeneration Template, which is a CG scaffold which promotes wound healing when combined with a temporary silicone sheet to maintain a moist wound environment and to act as a barrier.^25,26^ While this scaffold has shown success in the treatment of burn injuries^27^ and has shown some promise for DFUs,^25^ the silicone sheet itself is not antimicrobial and does not directly deal with infections and inhibit bacterial growth. We propose the addition of a biomimetic antimicrobial epidermal barrier layer to a similar CG scaffold to prevent wound infection and enhance healing.

While traditional wound dressings, including gauze, lint, bandages, and cotton wool, simply act as a cover for the wound,^28^ modern wound dressings have been developed to promote tissue regeneration and healing, rather than just covering of the wound.^29^ Chitosan, a naturally occurring bioactive polysaccharide, has been identified as an antimicrobial biomaterial which shows promise in a range of applications including as wound dressings^30,31^ and as a local drug delivery system to prevent wound infection.^32^ Although the exact mechanism of chitosan’s antimicrobial activity is not fully known, there are some generally accepted mechanisms described within the literature.^33−35^ The most accepted mechanism of action against bacteria is chitosan’s ability to disrupt the cell membrane through interaction of chitosan’s cationic amine groups with anionic moieties at the cell surface. Chitosan has been shown to exhibit effective antimicrobial activity against *Staphylococcus aureus*, the most common bacteria found in infected DFUs,^36^ with a minimum growth inhibitory concentration (MIC) of 20 ppm.^37^ In addition, with the number of infections caused by multidrug-resistant bacteria increasing across the world,^38^ it is important to note that chitosan maintains its antimicrobial properties against methicillin-resistant *S. aureus*.^39^ We propose that this makes it an excellent candidate for an antimicrobial film to prevent wound infection.

This paper thus describes the development and *in vitro* assessment of a bilayered biomimetic antimicrobial scaffold to promote healing of complex wounds such as DFUs. This scaffold consists of a dermal CG porous scaffold layer to enhance wound healing by supporting angiogenesis and an epidermal antimicrobial collagen/chitosan (CCh) film layer for the prevention of infection. The fabrication process was first optimized to produce a bilayered scaffold that mimicked the structure of the epidermal and dermal layers of the skin while maintaining adhesion strength between scaffold layers. The effects of physical and chemical cross-linking techniques to increase the structural stability of the bilayered scaffold were then studied through mechanical testing, scaffold swelling ability, and degradation studies. Following this, biological characterization assessed the antimicrobial activity of the CCh film against *S. aureus* by studying the inhibition of bacterial growth on the film and as a barrier to infiltration of the bacteria into the dermal CG layer. The ability of the scaffold to support re-epithelialization on the surface of the CCh film was investigated through seeding of human keratinocytes, and finally, the scaffold’s ability to support angiogenesis was investigated by assessing the response of human vascular cells.

## 2 Methods

### 2.1 Bilayered Scaffold Fabrication

**Figure 1:**
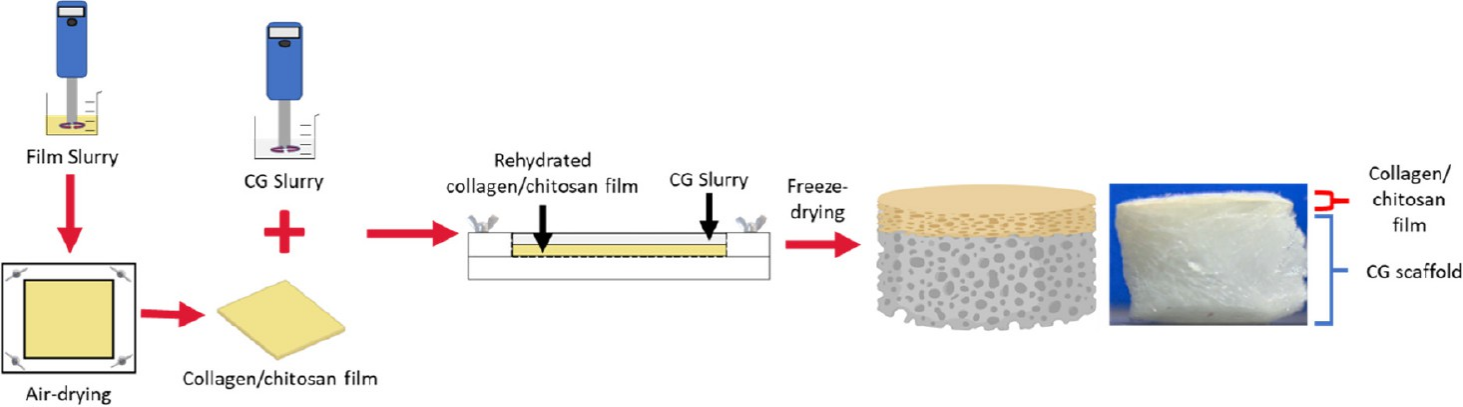
Fabrication process of the bilayered scaffold with a collagen chitosan epidermal film layer and a dermal CG scaffold layer. The fabrication process begins with the homogenization of a CCh slurry, followed by air-drying the slurry in a mold to produce a CCh film. The film is rehydrated, and a previously homogenized CG slurry is added before combining the two layers via freeze-drying to produce the bilayered scaffold.

Fabrication of the bilayered scaffold is a multistep process that involves the fabrication of an epidermal collagen/chitosan film and the subsequent addition of the CG slurry that is combined with said film via lyophilization (Figure 1).

#### 2.1.1 Fabrication of the Collagen/Chitosan Film

A solution of chitosan (Sigma-Aldrich, USA, medium molecular weight (190–310 kDa), 75–85% deacetylation) (0.75% w/v, chosen to ensure appropriate viscosity for handling) and microfibrillar type I collagen (1% or 0.5% w/v) (Integra Life Sciences, USA) in 0.5 M acetic acid was made. The solution was stored at 4 °C for 24 h to facilitate homogenization prior to blending using an Ultra-Turrax T 25 homogenizer (IKA, Germany) at 15 000 rpm at 4 °C. The film solution was then degassed under centrifuge at 4 °C, 1800 rpm for 25 min. The degassed film solutions were then pipetted into 6 × 6 cm aluminum molds fixed onto Teflon plates and left to air-dry for approximately 60 h.^40^

#### 2.1.2 CG Slurry Fabrication

A CG slurry for fabrication of the dermal layer was prepared as previously described.^41^ Briefly, a 0.5% w/v of microfibrillar, type I collagen isolated from bovine tendon (Integra Life Sciences, USA) and 0.05% w/v chondroitin-6-sulfate (C6S) isolated from shark cartilage (Sigma-Aldrich, Germany) solution was made in acetic acid (0.05 M) by blending using an Ultra-Turrax T 25 homogenizer (IKA, Germany) at 15 000 rpm at 4 °C. The CG slurry was then degassed under vacuum (∼5 Torr) at room temperature to remove air bubbles caused by blending. The CG slurry was stored at 4 °C until use.

#### 2.1.3 Combination of Epidermal Collagen/Chitosan Film with a Dermal CG Scaffold Layer

A lyophilization process was used to combine the antimicrobial CCh films (“epidermal layer”) with the CG scaffold (“dermal layer”) to create a bilayered scaffold. CCh films were hydrated in 0.5 M acetic acid for 5 min, 30 min, or 1 h, as this affects the final morphology of the epidermal film layer following lyophilization. The hydrated films were then placed on aluminum freeze-drying plates, and 6 × 6 cm aluminum molds were placed on top of the CCh films, ensuring that the film edges reached slightly under the borders of molds to prevent any gaps occurring between the films and the mold edges. Portions of the degassed CG slurry were pipetted onto the hydrated films, and the film/CG slurry structure was frozen to a final temperature of −10 °C at a rate of 1 °C min^–1^,^41^ which was maintained for 60 min, and then sublimated under vacuum (200 mTorr) at 0 °C for 29 h. The bilayered scaffold with the 0.5% collagen, 0.75% chitosan film (0.5% film) will be referred to as the 0.5% bilayered scaffold, and the bilayered scaffold with the 1% collagen, 0.75% chitosan film (1% film) will be referred to as the 1% bilayered scaffold. Scaffold layer composition can be seen in Table 1.

**Table 1:** Composition of Scaffold Layers in the 0.5% and 1% Bilayered Scaffolds

	1% bilayered scaffold	0.5% bilayered scaffold
dermal layer	0.5% collagen I, 0.05% C6S	0.5% collagen I, 0.05% C6S
epidermal layer	1% collagen I, 0.75% chitosan	0.5% collagen I, 0.75% chitosan

#### 2.1.4 Enhancement of Scaffold Structural Properties via Cross-Linking

Following fabrication, bilayered scaffolds were cross-linked by either physical or chemical means to assess which treatment resulted in the biggest increase in structural stability. Scaffolds underwent dehydrothermal (DHT) cross-linking @105 °C for 24 h using a vacuum oven (Vacucell, MMM Group, Germany) at 0.05 Barr.^22^

Chemical cross-linking was performed using 1-ethyl-3-(3-dimethylaminoopropyl)carbodiimide (EDAC) and *N*-hydroxysuccinimide (NHS) (Sigma-Aldrich, Germany). Scaffolds were hydrated in 70% EtOH (aq) for 1 h prior to cross-linking. EDAC (6 mmol per gram of collagen to be cross-linked) and *N*-hydroxysuccinimide NHS (2.5 M ratio of EDAC:NHS) were added to dH_2_O and were mixed using a vortexer (Vortex-Genie 2, Scientific Industries, USA). The scaffolds were transferred to the EDAC, NHS solution and allowed to cross-link at room temperature for 2 h. To remove cross-linking agents and to sterilize the scaffolds before cell culture experiments, the scaffolds were washed 3× (5 min each wash) in 70% ethanol, followed by 3× washes (5 min each wash) in PBS to remove residual ethanol.

### 2.2 Structural Characterization of the Bilayered Scaffold

#### 2.2.1 Measurement of Interfacial Adhesion Strength between Layers of the Bilayered Scaffold

Adhesion strength between layers of the bilayered scaffold was measured to ensure that delamination between the dermal and epidermal layers did not occur. Using Loctite Universal Super Glue (Radionics, Dublin), bilayered scaffolds were fixed, top and bottom, to aluminum SEM stubs. The adhesive was allowed to dry for 5 min before testing. The samples were loaded into the mechanical testing machine (Zwick/Roell, Ulm, Germany), and the maximum force (*F*_max_) was measured during delamination (20% strain min^–1^) between the scaffold layers using a 50 N loading cell.

#### 2.2.2 Scaffold Ultrastructure Characterization

The scaffold ultrastructure was observed using scanning electron microscopy (SEM) to determine the topography of the epidermal CCh films following lyophilization and cross-linking treatment, the pore structure within the bilayered scaffolds, as well as identification of the interface between the epidermal and dermal layers of the scaffold. Dry scaffold samples were cooled inside an Eppendorf tube and snap frozen in liquid nitrogen to allow cutting. The scaffolds were cut using a microtome blade to expose a transverse cross section. The scaffold samples were fixed onto SEM stubs using carbon tape and imaged at the Advanced Microscopy Lab facility (Trinity College Dublin). The samples were sputter-coated with a ∼ 5 nm layer of gold/palladium (80:20) using a Cressington 108 auto sputter-coater. Imaging was then carried out using a Zeiss Ultra FE-SEM at an accelerating voltage of 3 kV, and images were acquired at varying magnifications.

#### 2.2.3 Pore-Size Analysis of the Dermal CG Scaffold Layer

Pore-size analysis was carried out as previously described.^41^ Briefly, CG scaffolds were embedded in JB-4 methacrylate (JB-4 embedding kit, Polysciences, Germany) and stained with toluidine blue (Sigma-Aldrich, Ireland) to determine the average pore size. CG scaffolds were prehydrated in PBS for 1 h before fixation in 10% formalin (Sigma-Aldrich, Ireland) overnight. Dehydration of the scaffolds was then carried out with two dH_2_O washes, followed by consecutive washes in increasing concentrations of EtOH (aq) up to 100% EtOH. Following dehydration, samples were equilibrated and embedded according to the manufacturer’s instructions. Scaffolds were then sectioned (7 μm thickness) using a Leica RM2255 rotary microtome at three different depths within the scaffold and stained using a 0.5% toluidine blue solution. The sections were imaged using a Nikon 90i optical microscope (Japan), and pore size was analyzed using ImageJ software.

#### 2.2.4 Compressive Modulus

The compressive modulus was measured as a measurement of scaffold stiffness, which influences structural stability. Eight millimeter diameter scaffold samples were cut using a hole puncher. These samples were soaked in PBS for 1 h prior to testing. Using a mechanical testing machine (Zwick/Roell, Ulm, Germany) with a 5 N loading cell, the samples underwent wet (submerged in PBS) compressive testing up to 10% strain.

#### 2.2.5 Swelling Ratio

The scaffold swelling ratio measures the scaffold’s ability to retain moisture. The scaffold swelling ratio was determined by weighing dry scaffolds (8 mm diameter) (*d*), immersing these scaffolds in PBS (2 h, 37 °C), removing excess PBS from the surface, and reweighing the wet scaffolds (*w*). The swelling ratio was calculated according to the following equation:^42^
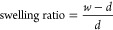
1

#### 2.2.6 Degradation Behavior

Analysis of the degradation behavior of the scaffold indicates the stability of the scaffold within the highly proteolytic wound environment. Scaffolds (8 mm diameter) were hydrated in PBS for 1 h. Excess liquid was removed from the surface of the scaffolds, and the scaffolds were weighed (*t* = 0). For hydrolytic degradation, scaffolds were kept in PBS at 37 °C. For enzymatic degradation, the scaffolds were kept in collagenase solution (from *clostridium histolyticum*, Type-F, ≥2.0 FALGPA units/mg solid) (2 mL, 0.05 mg/mL) (Sigma-Aldrich, Ireland) at 37 °C. Scaffolds had excess liquid removed from the surface before being weighed at specific time points.

### 2.3 Biological Characterization of the Bilayered Scaffold

#### 2.3.1 Antimicrobial Activity of the Collagen/Chitosan Films

The antimicrobial activity of the CCh films was tested against *Staphylococcus aureus* (*strain Newman*) using live/dead staining and imaging.^43^ Using a 1% collagen-only film as a control, 8 mm diameter film discs were placed in 24-well plates and were incubated (24 h, 37 °C) in a bacterial broth solution (1 mL) containing 1 × 10^6^ CFU. Following incubation, the bacterial broth was removed, and three washes with PBS were carried out to remove any unattached bacteria. Bacteria were then stained using a LIVE/DEAD Viability Kit (Thermo Fisher, Ireland), according to the manufacturer’s instructions. The stained bacteria on the films were imaged using a Carl Zeiss LSM 710 confocal microscope, and bacterial viability was calculated based on the percentage of the area that the bacteria covered on the film which were alive. Images were analyzed using ImageJ software.

#### 2.3.2 Barrier Function Analysis of the Collagen/Chitosan Film against Bacteria

The ability of the epidermal CCh film layer to act as a barrier to prevent bacteria from infiltrating to the wound was examined. This was done by adding 50 μL of a bacterial broth (*S. aureusstrain Newman*, 7 × 10^7^ CFU mL^–1^) on top of the bilayered scaffold and a CG-only scaffold control (i.e., without the CCh film layer). These scaffolds were incubated for 24 h at 37 °C and then fixed overnight at 4 °C with a 10% formalin solution. The scaffolds were then incubated (3 min, RT) with 0.1% Triton X-100 (Sigma-Aldrich, Ireland) and washed again (3× washes) with PBS. Nuclei staining was carried out using Hoechst 33342 (Invitrogen, Thermo Fisher Scientific, USA) (1 mL per scaffold, 1:5000 PBS dilution), and the scaffolds were incubated (20 min, RT) while covered in aluminum foil. The scaffolds were then washed (3× washes) and stored in PBS. Following staining, the scaffolds were imaged using a Carl Zeiss LSM 710 confocal microscope, with an N-Achroplan 10× (numerical aperture (N.A.) 0.3), and image analysis was carried out using ImageJ.

#### 2.3.3 Culture of Human Keratinocytes on the Epidermal CCh Film Layer

The ability of the epidermal CCh film to act as a surface for re-epithelialization of the wound was tested through culture of human keratinocytes (HaCaT; cell line derived from histologically normal skin keratinocytes) on the films. HaCaTs were cultured in low glucose (1 mg/mL) Dulbecco’s Modified Eagle Medium (DMEM) (Sigma-Aldrich, Ireland) supplemented with 10% FBS until they were 80–90% confluent. They were then passaged, and 15 000 cells were seeded onto the center of each film (8 mm diameter). One milliliter of DMEM was then added to each film well, and the films were incubated (37 °C, 5% CO_2_).

#### 2.3.4 3D Culture of Vascular Cells on the Dermal CG Scaffold Layer

Vascular cells were differentiated from human-induced pluripotent stem cells (hiPSCs) and sorted into two populations of CD31-positive cells (endothelial cells—iECs) and CD31-negative cells (stromal cells—iSCs), as previously described.^44^ Both cell populations (iECs and iSCs) were cultured in gelatin-coated flasks in EGM-2 (Promocell, Germany). When cells were approximately 80–90% confluent, they were passaged and seeded on CG scaffolds. The CG scaffolds were seeded with a 1:1 ratio of iEC:iSC. Scaffolds were seeded with 250 000 total cells pipetted onto the center of each scaffold. Five hundred microliters of fresh EGM2 was then added to each scaffold well, and the scaffolds were incubated overnight (37 °C, 5% CO_2_). The seeded scaffolds were then placed into new wells, and the media (EGM2) were replaced with 500 μL of fresh media. The scaffolds were fed every 2–3 days with fresh media (EGM2).

#### 2.3.5 Analysis of Cell Metabolic Activity

To analyze and compare cell metabolic activity between the different cross-linked scaffolds (NXL, DHT, EDAC), different film compositions (0.5% and 1% films), and different film cross-linking treatments (NXL, DHT, EDAC), an AlamarBlue assay (Invitrogen, Thermo Fisher Scientific, USA) was performed according to the manufacturer’s instructions. Briefly, media from the CG scaffolds/CCh films seeded with iECs and iSCs and HaCaTs, respectively, were replaced with fresh EGM2 media supplemented with 10% AlamarBlue. Following incubation, fluorescence intensity was measured (λ_excitation_ = 540–570 nm, λ_emission_ = 580–610 nm) using a plate reader (Tecan Group Ltd., Switzerland). Cell metabolic activity was measured at day 1, 3, and 7 after seeding for the dermal CG scaffolds and after day 1 and 3 for the epidermal CCh films.

#### 2.3.6 Fluorescence Staining and Imaging of Cell-Seeded Scaffolds/Films

To evaluate the effect of cross-linking (NXL, DHT, EDAC) on the ability of the dermal CG scaffold layer to support the iEC, iSC coculture and the forming of a vascular structure in 3D and the ability of the epidermal CCh film layer to support the proliferation of keratinocytes, the CG scaffolds/CCh films were fluorescently stained. Cell-seeded scaffolds were fixed overnight at 4 °C in 10% formalin (Sigma-Aldrich, Ireland) after 7 days of culture, and CCh films were fixed for 30 min in 10% formalin after 3 days of culture. For cytoskeleton staining, the scaffolds/films were incubated in Phalloidin-Atto 488 (Sigma-Aldrich, Ireland) (1:600 in PBS) (1 h, RT). Nuclei staining was carried out using DAPI (500 μL per scaffold, 1 μg/mL in PBS) (20 min, RT). Following staining, the scaffolds/films were imaged using a Carl Zeiss LSM 710 confocal microscope, with an N-Achroplan 10× (N.A. 0.3) and W Plan-Apochromat 20× (N.A. 1.0) objective. Image analysis was carried out using ImageJ.

### 2.4 Statistical Analysis

Results were analyzed using GraphPad Prism software version 8.0.2 (San Diego, CA) by carrying out a one-way ANOVA when more than one treatment was compared and a two-way ANOVA when more than one treatment was compared across two factors, both followed by Tukey’s post hoc test. All tests were carried out in triplicate with multiple technical repeats, and error bars are expressed as ±SD.

## 3 Results

### 3.1 Bilayered Scaffold Fabrication

#### 3.1.1 Shortening Collagen/Chitosan Film Hydration Time Led to a Flatter, Filmlike Morphology without Impacting Interfacial Adhesion Strength

**Figure 2:**
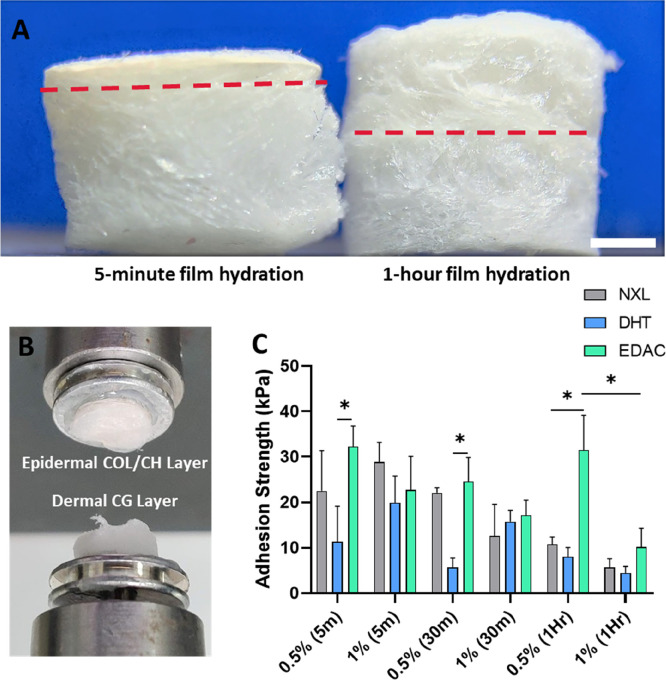
Reduction in swelling time of the epidermal CCh film resulted in a flatter, more compact film layer while maintaining good adhesion strength with the dermal CG scaffold layer, with the EDAC cross-linked 0.5% bilayered scaffold with a 5 min swelling time having the highest adhesion strength of all groups. (A) Cross section of the bilayered scaffold showing a thinner, more compact epidermal CCh film layer as a result of reducing the hydration time prior to freeze-drying to 5 min (1 mm thickness) from 1 h (4 mm thickness). Red line indicates the interface between the dermal CG scaffold layer (bottom) and the epidermal CCh film (top). Scale bar = 200 μm. (B) Setup of the measurement of adhesion between the layers of the bilayered scaffold showing delamination at the interface between the layers. (C) Adhesion strength between the epidermal CCh film layer and the dermal CG scaffold layer of the bilayered scaffold, with the highest adhesion strength seen in the EDAC cross-linked 0.5% bilayered scaffold at 32.3 kPa. Data shown are mean ± SD. * denotes a significance of *p* < 0.05 (two-way ANOVA; Tukey’s posthoc test) (*N* = 3).

An epidermal antimicrobial CCh film and a dermal CG porous scaffold were successfully combined via initial hydration of the film and subsequent lyophilization of the hydrated film with an overlaid CG slurry to generate the bilayered scaffold (Figure 1). The initial hydration time of the film impacted the final morphology of the epidermal CCh film layer, as shown in Figure 2A. When hydrating the film for 1 h, the resulting CCh layer had a thickness of approximately 4 mm. Reducing the hydration time of the film to 5 min still allowed for adhesion between the layers of the bilayered scaffold but reduced the CCh film thickness to approximately 1 mm, thus preserving its barrier function. Delamination tests (Figure 2B) were carried out to measure the effect of hydration time on adhesion strength between each of the scaffold layers, and it was found that the EDAC cross-linked 0.5% bilayered scaffold with a 5 min hydration time had the highest adhesion strength (32.3 kPa ± 4.5) across all scaffolds (Figure 2C). However, an increase in the hydration time to 30 min or 1 h did not significantly impact the adhesion strength. A 5 min film hydration time preserved the filmlike morphology of the epidermal layer without impacting the adhesion strength between the epidermal CCh and dermal CG layers of the scaffold. In all tests, delamination occurred at the interface between the dermal CG and epidermal CCh layers.

### 3.2 Structural Characterization of the Bilayered Scaffold

#### 3.2.1 The Bilayered Scaffold Consists of a Porous CG Scaffold Layer with a Flat, Continuous CCh Film Layer

**Figure 3:**
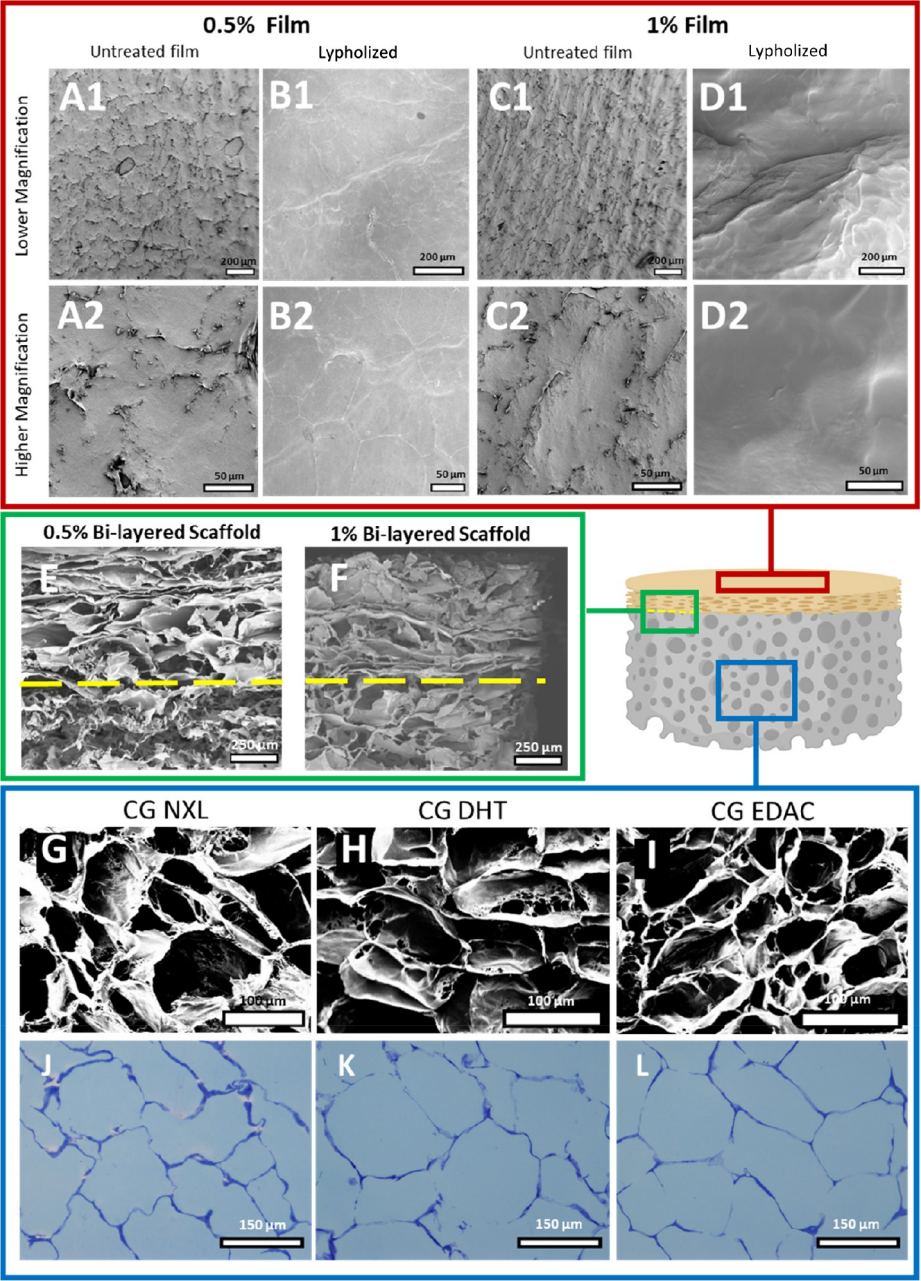
SEM imaging showing that the bilayered scaffold maintains its filmlike topography in the epidermal CCh film layer following freeze-drying as well as the porous structure of the dermal CG layer. (A–D) Images of the epidermal CCh film surface of the bilayered scaffold in the (A1,2) 0.5% bilayered scaffold before freeze-drying, (B1,2) 0.5% bilayered scaffold following freeze-drying, (C1,2) 1% bilayered scaffold before freeze-drying, and (D1,2) 1% bilayered scaffold following freeze-drying. (A1–D1) Scale bar = 200 μm. (A2–D2) Scale bar = 50 μm. (E–F) Images of the cross sections of the (E) 0.5% bilayered scaffold and (F) 1% bilayered scaffold showing the interface (yellow dashed lines) between the epidermal CCh film layer (above the line), which has a denser filmlike structure, and the highly porous dermal CG scaffold layer (below the line). Scale bar = 250 μm. (G, H, and I) Highly porous structure of the NXL, DHT, and EDAC cross-linked dermal CG scaffold layer of the bilayered scaffold, respectively. Scale bar = 100 μm. (J, K, and L) Dermal CG scaffold layer pore size was measured to be 148 ± 7, 146 ± 11, and 150 ± 5 μm in the NXL, DHT, and EDAC CG scaffolds, respectively, as determined by toluidine blue staining (*N* = 3). Scale bar = 150 μm.

Scanning electron microscopy (SEM) was used to examine the ultrastructure of both layers and the interface of the bilayered scaffold (Figures 3A–I). Prior to rehydration and lyophilization, the CCh films had a flat, continuous topography (Figures 3A and C). This topography was maintained following incorporation into the bilayered scaffold (Figures 3B and D). Imaging of the cross section of the bilayered scaffold (Figures 3E and F) shows the interface between the epidermal layer, with a dense barrier-like appearance, and the dermal layer, showing a highly porous structure (indicated by yellow dashed lines). This confirmed that the scaffold has two distinct layers. Examining the cross section of the NXL, DHT, and EDAC cross-linked dermal CG scaffold (Figures 3G–I) layer showed a highly porous structure. Following toluidine blue staining of the scaffolds (Figures 3J–L), the average pore sizes of the NXL, DHT, and EDAC cross-linked scaffolds were determined to be 148 ± 7, 146 ± 11, and 150 ± 5 μm, respectively (Figure S1), with no significant difference detected between the groups, and were consistent with pore sizes previously reported.^41^ This examination of the bilayered scaffold structure shows that the epidermal CCh film surface maintains a flat, continuous morphology, which is important to its barrier function. In contrast, the dermal CG scaffold has a highly porous dermal CG layer, which will support infiltration of cells during the angiogenic and wound healing processes.

#### 3.2.2 EDAC Cross-Linking of the Bilayered Scaffold Results in the Greatest Structural Stability

**Figure 4:**
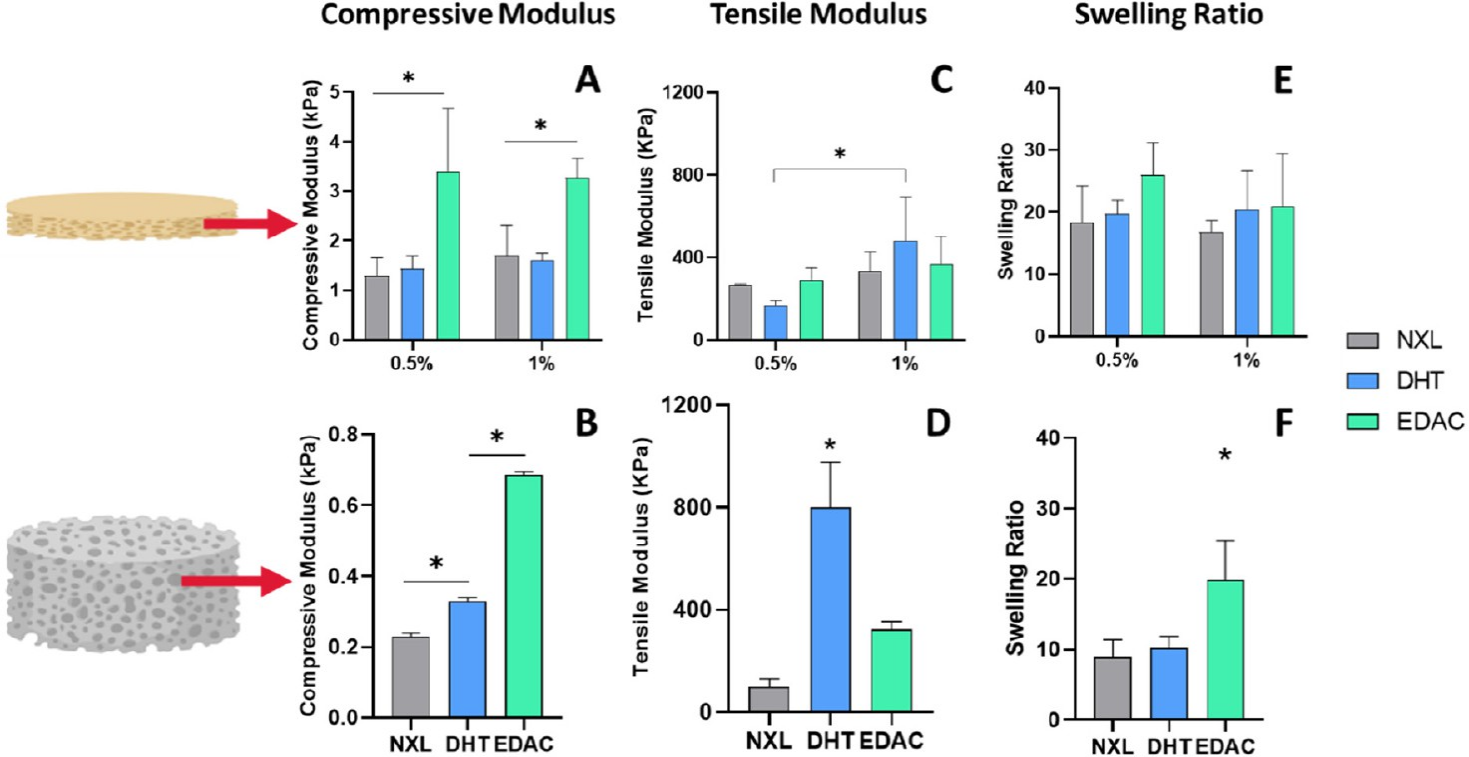
Structural characterization of the individual layers of the bilayered scaffold showing that the 0.5% EDAC cross-linked bilayered scaffold had the highest mechanical properties in all tests but the tensile modulus. (A) Compressive modulus of the epidermal CCh film layer showed the EDAC cross-linked films had significantly higher compressive moduli than the NXL and DHT cross-linked films. (C) The tensile modulus of the films did not significantly change following cross-linking; however, the 1% collagen, 0.75% chitosan film had a significantly higher tensile modulus than the DHT cross-linked 0.5% collagen, 0.75% chitosan film. (E) Swelling ratio of the films showed an increasing trend following cross-linking, but the change was not significant. (B) The compressive modulus of the dermal CG scaffold significantly increased following cross-linking, with EDAC cross-linking giving a significantly higher modulus than DHT cross-linking. (D) The tensile modulus of the dermal CG scaffold increased, but not significantly, following EDAC cross-linking. DHT cross-linking resulted in a significant increase in tensile modulus. (F) The swelling ratio of the dermal CG layer increased significantly following EDAC cross-linking. Data shown are mean ± SD. * denotes a significance of *p* < 0.05 (two-way ANOVA; Tukey’s posthoc test) (*N* = 3).

Further mechanical testing was carried out to determine the effect of cross-linking and film composition on the structural stability of the bilayered scaffold. First, looking at the epidermal CCh films (Figures 4A, C, and E), the highest compressive modulus (Figure 4A) was seen in the EDAC cross-linked films, with the 0.5% and 1% films having a compressive modulus of 3.4 ± 1.3 and 3.3 ± 0.4 kPa respectively, which was significantly higher than for the NXL and DHT cross-linked groups. The tensile modulus of the films (Figure 4C) was increased following cross-linking, but no significant increase was seen within the 0.5% or 1% groups when comparing NXL and DHT/EDAC cross-linked films. Similarly, the swelling ratio of the films (Figure 4E), which demonstrates their ability to retain moisture, was unchanged following DHT/EDAC cross-linking.

When looking at the dermal CG scaffold layer, the compressive modulus (Figure 4B) significantly increased following cross-linking. The EDAC cross-linked CG scaffold had a significantly higher compressive modulus (0.68 ± 0.01 kPa) than both the DHT and NXL groups. The tensile modulus of the CG scaffold layer (Figure 4D) increased following both cross-linking treatments versus the NXL scaffold, with the DHT scaffold having the highest tensile modulus (799 ± 178 kPa). The swelling ratio of the CG scaffold layer (Figure 4F) was significantly increased following EDAC cross-linking (19.9 ± 5.6). Taken together, these results suggest that the 0.5% EDAC cross-linked bilayered scaffold has the highest structural stability of the scaffolds tested.

#### 3.2.3 The EDAC Cross-Linked Bilayered Scaffold Shows the Greatest Resistance to Degradation

**Figure 5:**
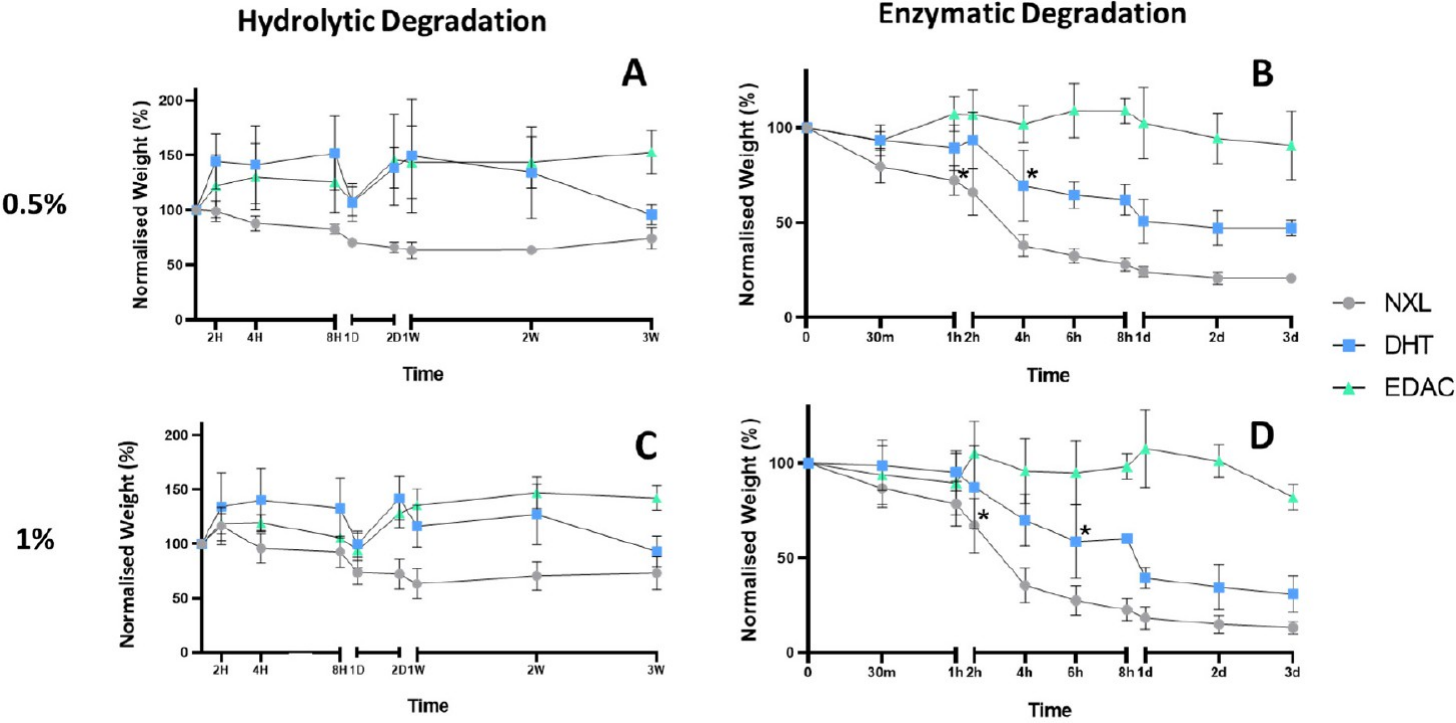
EDAC cross-linked bilayered scaffolds show resistance to enzymatic degradation by collagenase. (A) Measuring the weight of the 0.5% bilayered scaffolds over time during incubation in PBS at 37 °C showed that the EDAC cross-linked scaffold maintained its weight better than its DHT and NXL counterparts, but no significant degradation was seen in any cross-linking group after 3 weeks. (B) Measuring the weight of the 0.5% bilayered scaffolds over time during incubation in collagenase (0.05 mg mL^–1^) at 37 °C showed that the EDAC cross-linked scaffold maintained its weight while its DHT and NXL counterparts showed significant degradation at *t* = 4 and 1 h, respectively. (C) Measuring the weight of the 1% bilayered scaffolds over time during incubation in PBS at 37 °C showed that the EDAC cross-linked scaffold maintained its weight better than its DHT and NXL counterparts, but no significant degradation was seen in any cross-linking group after 3 weeks. (D) Measuring the weight of the 1% bilayered scaffolds over time during incubation in collagenase (0.05 mg mL^–1^) at 37 °C showed that the EDAC cross-linked scaffold maintained its weight while its DHT and NXL counterparts showed significant degradation at *t* = 6 and 2 h, respectively. Data shown are mean ± SD. * denotes significance, *p* < 0.05 (two-way ANOVA; Dunnett’s multiple comparison test) (*N* = 3).

Having identified that EDAC cross-linking of the bilayered scaffold resulted in the highest structural stability, we sought to test the effects of cross-linking treatment on the scaffold’s resistance to degradation. The hydrolytic degradation (PBS) of the 0.5% and 1% bilayered scaffolds is shown in Figures 5A and 5C, respectively. Over 3 weeks, no significant weight loss was detected in any of groups. Having seen no degradation in hydrolytic conditions, the enzymatic degradation of the scaffolds was measured. The enzymatic degradation (collagenase, 0.05 mg mL^–1^) of the 0.5% and 1% bilayered scaffolds is shown in Figures 5B and 5D, respectively. There was no significant degradation (loss of scaffold weight) seen in either of the EDAC cross-linked bilayered scaffolds after 3 days. The NXL and DHT cross-linked 0.5% bilayered scaffolds saw 79.2 ± 1.6% and 52.8 ± 4.3% degradation, respectively, after 3 days. The NXL and DHT cross-linked 1% bilayered scaffolds saw 86.7 ± 3.2% and 69.0 ± 9.7% degradation, respectively, after 3 days. It is important to note that bilayered scaffold weight will not reach zero due to the presence of chitosan, which is unaffected by collagenase. These degradation studies show that EDAC cross-linked bilayered scaffolds have the greatest resistance to enzymatic degradation.

### 3.3 Biological Characterization of the Bilayered Scaffold

#### 3.3.1 The Epidermal CCh Films Inhibits the Growth of *S. aureus*, While Also Preventing Infiltration of the Bacteria into the Dermal CG Scaffold Layer

**Figure 6:**
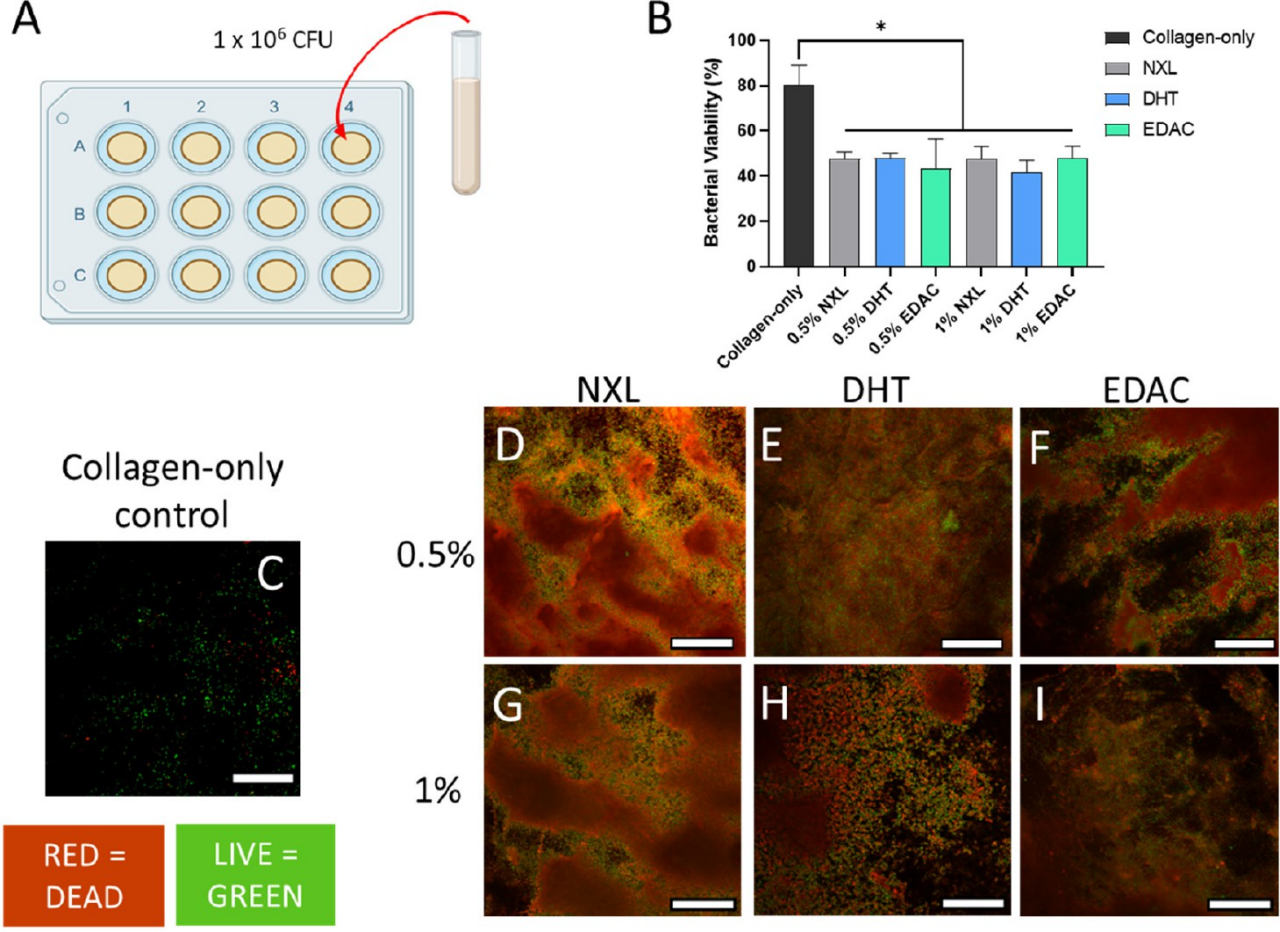
Epidermal CCh films demonstrate antimicrobial activity against *S. aureus*, which is maintained following cross-linking treatment. (A) Films were incubated for 24 h in a bacterial broth containing 1 × 10^6^ cells. (B) The CCh films significantly reduced the viability of *S. aureus* when compared to a collagen-only film. Data shown are mean ± SD. * denotes significance, *p* < 0.05 (one-way ANOVA; Tukey’s posthoc test) (*N* = 3). (C) Imaging of live and dead bacteria on the collagen-only film. (D–I) Imaging of live and dead bacteria on the NXL, DHT, and EDAC cross-linked (D–F) 0.5% collagen, 0.75% chitosan films and (G–I) 1% collagen, 0.75% chitosan films (red = dead, live = green).

The antimicrobial activity of the CCh films against *S. aureus* was analyzed by incubating the films in a bacterial broth for 24 h (Figure 6A) and then assessing bacterial viability using LIVE/DEAD staining (Figures 6B–I). Bacterial viability was significantly reduced in both the 0.5% and 1% CCh films across all cross-linking treatments versus a collagen-only film control (Figure 6B). This can be seen with mostly live bacteria seen in green on the collagen-only control (Figure 6C) versus largely dead bacteria seen in red on the CCh films (Figures 6D–I).

**Figure 7:**
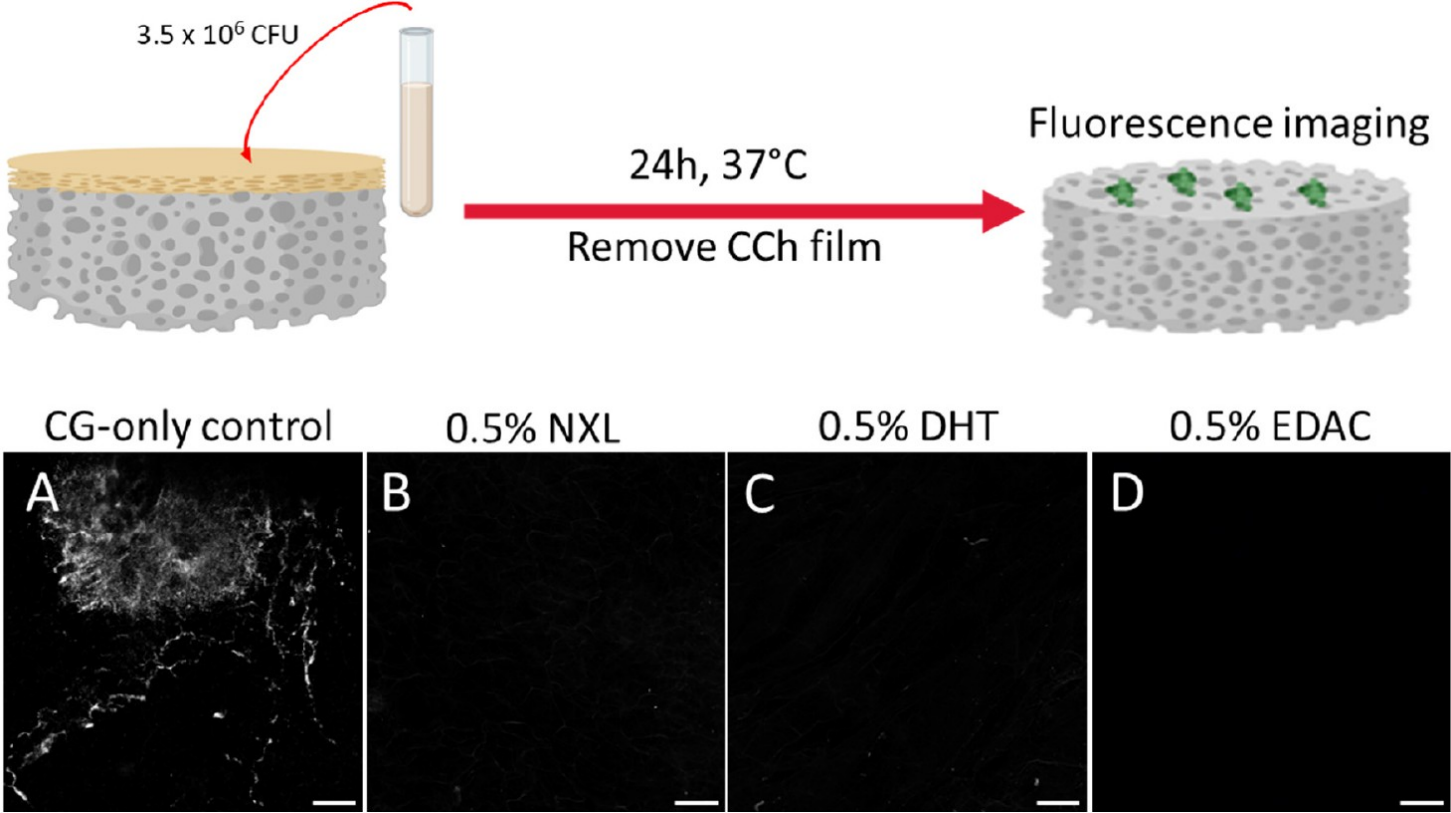
0.5% collagen, 0.75% chitosan film formed a successful barrier to bacteria to prevent infiltration into the dermal CG scaffold layer. (A) Imaging of DAPI-stained (white) *S. aureus* on a CG scaffold versus (B–D) CG scaffold layers that had a 0.5% CCh epidermal film which acted as a barrier to bacterial infiltration during bacterial seeding and was removed prior to imaging the stained dermal CG scaffold layer.

When assessing the ability of the CCh films to act as a barrier to the infiltration of bacteria, the 0.5% bilayered scaffold was the only scaffold brought forward for testing as it had the highest structural stability versus the 1% bilayered scaffold. It was demonstrated that the 0.5% CCh film in the 0.5% bilayered scaffold formed an effective barrier to prevent the infiltration of *S. aureus* into the dermal CG scaffold layer (Figure 7). The dermal CG-scaffold-only control (Figure 7A) showed an abundance of bacteria (white) on its surface, while the presence of the epidermal CCh film prevented nearly all bacterial infiltration to the dermal CG layer in the NXL and DHT 0.5% bilayered scaffolds (Figures 7B and C, respectively), and no bacteria was found in the EDAC cross-linked 0.5% bilayered scaffold dermal layer (Figure 7D) following removal of the film. Overall, this suggests that the epidermal CCh film can not only inhibit the growth of *S. aureus* but also acts as an effective barrier to prevent the infiltration of these bacteria into the dermal CG scaffold layer and wound environment. With higher structural stability, the 0.5% bilayered scaffold was determined as the best performing scaffold composition.

#### 3.3.2 The Epidermal CCh Film Layer Supports the Proliferation of Keratinocytes

**Figure 8:**
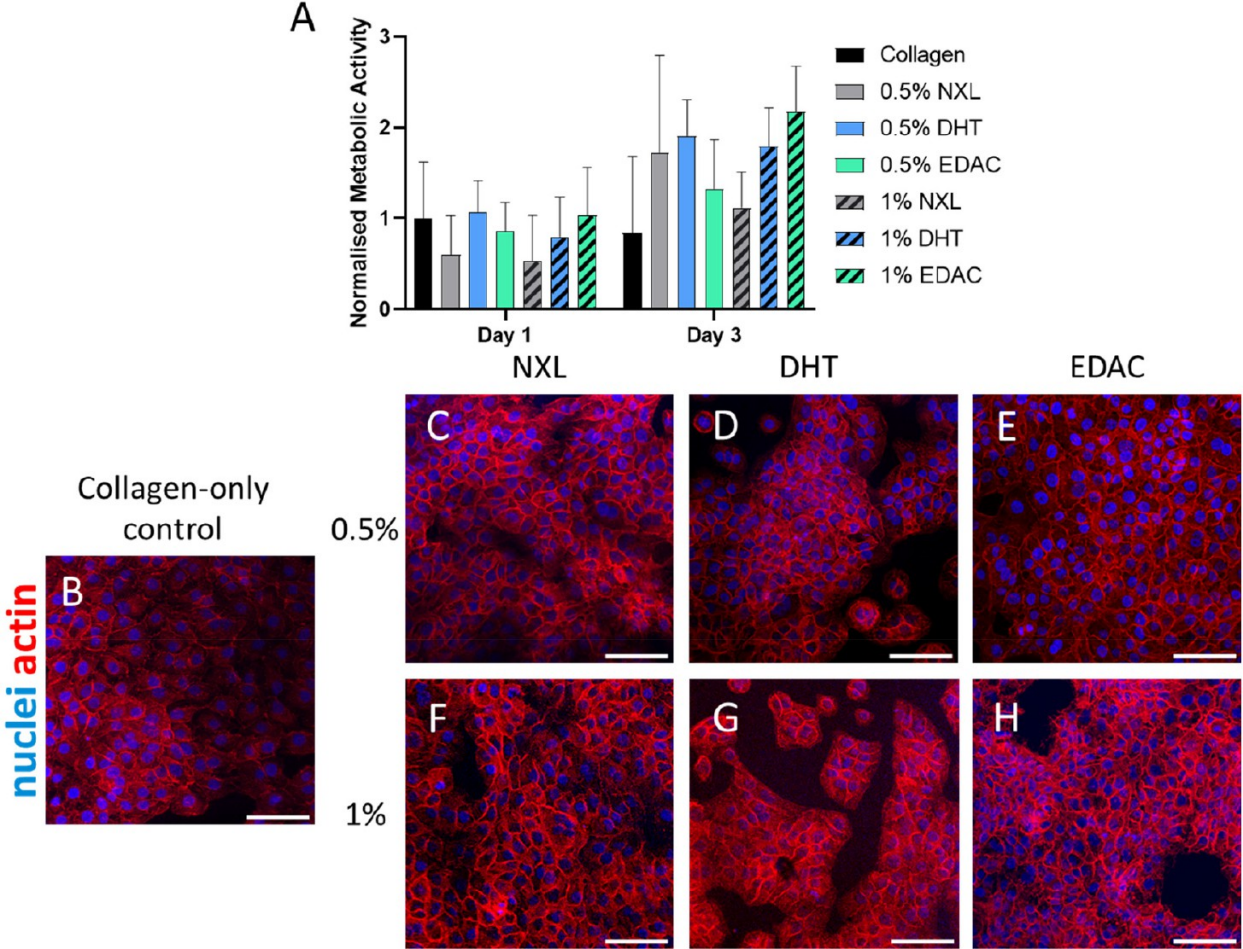
Epidermal CCh films support the proliferation of HaCaTs on its surface. (A) Viability of HaCaTs cultured on the epidermal CCh films was maintained after 3 days, regardless of cross-linking treatment, as shown by AlamarBlue assay normalized to the 1% collagen film control on day 1. Data shown are mean ± SD (two-way ANOVA; Tukey’s multiple comparisons test) (*N* = 4). (B–H) Fluorescent staining of the HaCaTs seeded on the (B) collagen-only control, (C–E) 0.5% collagen, 0.75% chitosan films, and (F–H) 1% collagen, 0.75% chitosan films shows cell populations with no signs of cytotoxicity. (Blue = dead, red = actin. Scale bars = 100 μm.)

Having demonstrated the epidermal CCh film layer’s antimicrobial activity, as well as its function as a barrier to infiltrating bacteria, we sought to show that the film also serves as a surface for re-epithelialization of the wound. Human keratinocytes (HaCaTs) were cultured on both the 0.5% and 1% CCh films, across all cross-linking treatments, for 3 days and compared to a collagen-only control. Cell growth was investigated using an AlamarBlue assay (Figure 8A), which showed no signs of toxicity in the HaCaTs over the 3 days of culture on the films. Fluorescent staining of HaCaTs was also carried out on the 0.5% CCh films (Figures 8C–E) and the 1% CCh films (Figures 8F–H) versus a collagen-only film control (Figure 8B). Dense monolayers of HaCaTs were seen on all film groups, regardless of composition or cross-linking treatment, showing no signs of cytotoxicity. These results indicate that the epidermal CCh layer of the scaffold can support the attachment and growth of human keratinocytes.

#### 3.3.3 The Dermal CG Scaffold Layer Supports the Proliferation of Vascular Cells

**Figure 9:**
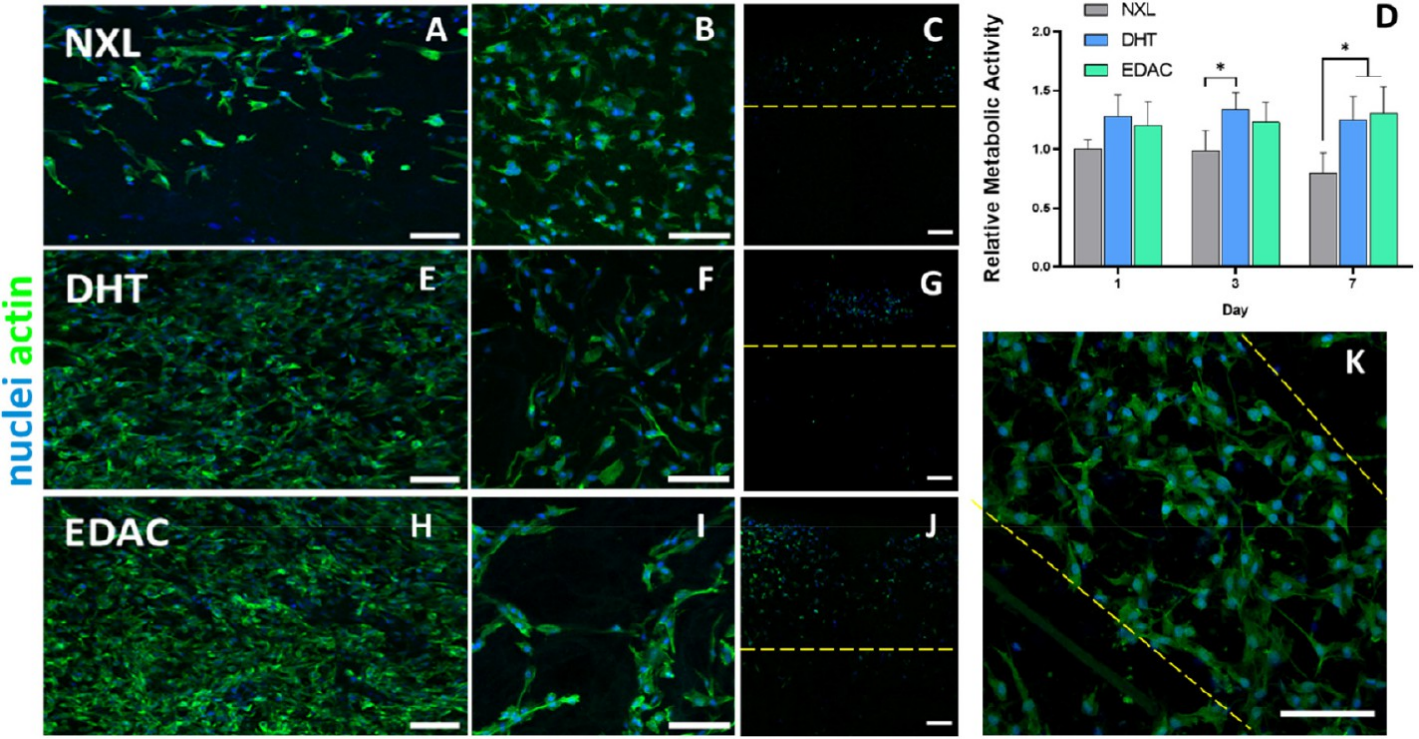
iECs and iSCs have increased proliferation and have more elongated cell bodies on cross-linked CG scaffolds. Fluorescence imaging of iECs and iSCs cocultured on (A, B, and C) NXL, (E, F, and G) DHT, and (I, J, and K) EDAC cross-linked CG scaffolds on day 7 of culture. First column (A, E, and H) shows increased proliferation on cross-linked scaffolds. Second column (B, F, and I) shows more elongated cell morphologies on cross-linked scaffolds versus NXL scaffolds, demonstrating better adhesion to the scaffold structure. Third column (C, G, and J) shows cellular infiltration into the scaffold as seen via cross section (highlighted within yellow dashed lines). (D) Proliferation of iECs and iSCs cultured on DHT cross-linked and EDAC cross-linked CG scaffolds was significantly higher than that of the NXL scaffold at day 7 when assessed by the AlamarBlue assay normalized to NXL day 1. Data shown are mean ± SD. * denotes a significance of *p* < 0.05 (two-way ANOVA; Tukey’s multiple comparisons test) (*N* = 4). (K) Formation of early vascular tubelike structures in EDAC cross-linked CG scaffolds (highlighted within yellow dashed lines). (Blue = nuclei, green = actin. Scale bars = 100 μm.)

To better understand the effects of cross-linking on the ability of the dermal CG layer of the bilayered scaffold to support angiogenesis, induced pluripotent stem cell derived endothelial and stromal cells (iEC and iSC) were cultured on the dermal CG layer of the scaffold. Fluorescent staining analysis was carried out on the NXL (Figures 9A–C), DHT (Figures 9E-G), and EDAC cross-linked (Figures 9H–K) CG scaffolds, along with an AlamarBlue proliferation assay (Figure 9D). Cells seeded on the cross-linked scaffolds showed enhanced proliferation as seen by the higher cell density (Figures 9E and H) versus on the NXL scaffold (Figure 9A). Elongated cell morphologies were also observed on the cross-linked scaffolds (Figures 9F and I), which was not observed on the NXL scaffold (Figure 9B). Cellular infiltration (Figures 9C, G, and J) was also enhanced on EDAC cross-linked scaffolds, with cells infiltrating further into the center of the scaffold when compared to the NXL and DHT scaffolds. The metabolic activity of the iEC and iSCs on the cross-linked scaffolds was also significantly higher at day 7 on the cross-linked versus NXL scaffolds (Figure 9D). Finally, in the EDAC cross-linked scaffold (Figure 9K), there are early signs of cells organizing into vascular tubelike structures observed after 7 days. This indicates that the EDAC cross-linked dermal CG scaffold layer can support the infiltration and proliferation of vascular cells to a greater extent than either the NXL or DHT cross-linked CG scaffolds.

## 4 Discussion

The objective of this work was the development of a biomimetic antimicrobial scaffold for enhanced wound healing. Building on previous existing expertise within our lab in the development of collagen-based scaffolds for tissue engineering applications,^16−19,45^ a bilayered antimicrobial scaffold was developed. Mimicking the structure of human skin, this scaffold consists of two distinct biomimetic layers, an epidermal CCh film layer to prevent wound infection and a dermal CG scaffold layer to promote angiogenesis. Structural characterization through assessment of the compressive and tensile modulus, swelling and degradation behavior, as well as analysis of the scaffold ultrastructure determined that the EDAC cross-linked 0.5% bilayered scaffold was the scaffold with the highest structural stability. *In vitro* analysis showed that the epidermal CCh film layer successfully inhibits the growth and infiltration of *S. aureus*, the most common bacterial isolate found in diabetic foot infections.^36^ Following the seeding of vascular cells, it was demonstrated that the dermal CG layer supported proliferation, and the early signs of vascular tubelike structures were observed, showing a capacity to support angiogenesis. Taken together, the results show that this bilayered scaffold can help overcome limitations of currently available biomaterial treatments for complex wounds by directly dealing with potential infections in the wound environment, while also promoting vascularization and ultimately directing successful tissue repair.

The bilayered scaffold was designed with the structure of native skin in mind, as well as to ensure the epidermal CCh film layer acted as a nonporous physical barrier to infiltrating bacteria. With the epidermal thickness of the plantar foot ranging from approximately 0.51 to 1.46 mm and the dermal thickness at roughly 3 mm,^46,47^ these values match closely to that of the epidermal CCh film and dermal CG scaffold at roughly 1 and 4 mm, respectively. A 5 min hydration of the CCh film prior to addition of the CG slurry resulted in a desired morphology with successful barrier formation in the epidermal layer. Furthermore, one of the most important characteristics when fabricating a bilayered material is the adhesion strength between the two layers of the material, and the scaffold developed herein demonstrates significant interfacial adhesion and resistance to delamination, which is often overlooked in studies developing multilayered materials for wound healing applications.^48−51^ This adhesion was particularly evident in the EDAC cross-linked 0.5% bilayered scaffold, even when reducing the hydration time of the film prior to lyophilization from 60 to 5 min. While the adhesion strength of the EDAC cross-linked 0.5% bilayered scaffold is lower than that of the stratum corneum in native skin (roughly 0.1–0.7 MPa),^52^ it was the strongest of all tested groups. Thus, if treatment using the scaffold is carried out with current standards of care, including pressure offloading, there should be very little risk of delamination occurring. We speculate that the improved adhesion between the layers comes from fibers in the CG slurry infiltrating the CCh film once added on top during fabrication. Swelling of the film layer is likely to enhance interdigitation of the fibrils between the epidermal film layer and the dermal CG scaffold layer. This is aided by the fact that the same solvent (acetic acid) is used to hydrate the film and that chitosan incorporation into collagen biomaterials is known to increase hydrophilicity^42^ and therefore allows for easy swelling of the film layer. Taken together, the fabrication process resulted in a bilayered scaffold that mimics the structure of native human skin while retaining the interfacial adhesion strength between scaffold layers.

As previously mentioned, the function of the epidermal CCh film in the bilayered scaffold is to act as an antimicrobial barrier for the wound. Lyophilization has been used widely in tissue engineering applications to create porous structures,^16−19,45^ and it was thus important to ensure that the top surface of the film was not porous following fabrication. Analysis of the ultrastructure of the film layer’s topography by SEM showed that the epidermal CCh layer maintained a nonporous, flat topography following rehydration and lyophilization. However, imaging of the cross section of the dermal CG layer showed retention of a highly porous structure. The highly porous architecture of these scaffolds allows for the infiltration and migration of cells throughout the structure and is important for the treatment of many diseased states, including promotion of angiogenesis/vascularization^17,18^ and wound healing.^53−57^ This demonstrates that the bilayered scaffold has a suitable structure, with the epidermal CCh layer providing a physical barrier and the dermal CG layer providing as well as a porous platform for cell infiltration and vascularization.

Having demonstrated a proof of concept and an initial design prototype, the next step was to optimize the structural properties of the bilayered scaffold. For applications in wound healing, the scaffold needs to withstand both compressive and tensile forces experienced during stretching of the surrounding skin, while also resisting delamination between layers. Mechanical characterization, through measurement of the compressive modulus, tensile modulus, swelling ratio, and resistance to degradation, determined that the EDAC cross-linked 0.5% bilayered scaffold has the highest structural stability. All cross-linking groups tested, for both the 1% and 0.5% bilayered scaffolds, had compressive moduli which fall between 0.5 and 1.1 kPa in the dermal CG scaffold layer, which is within the expected range for a single-layer CG scaffold.^22,57,58^ Scaffolds of similar composition currently exist on the market for wound healing applications, an example being the Integra Dermal Regeneration Template.^25,26^ Comparable mechanical stiffness in the dermal layer to products already on the market gives a good indication that these scaffolds have suitable mechanical properties and integrity for implantation in a diabetic wound environment.

For the scaffold to be successfully implanted into the wound in a clinical environment, it will need to be sutured in place at the wound’s edge and will likely endure tensile strain. When considering the practicality of suturing the bilayered scaffold into the wound, the epidermal CCh film will endure greater tensile forces as the suture thread goes through this layer. Both DHT and EDAC cross-linking resulted in increased tensile modulus in the epidermal CCh film layer, with no significant difference seen between the two treatments. Therefore, considering that EDAC cross-linking resulted in a significantly higher compressive modulus in the dermal CG layer, it was observed that, overall, EDAC cross-linking of the bilayered scaffold led to the highest structural properties.

A moist wound environment during wound healing allows for the maintenance of cellular activity, an increase in the breakdown of dead tissue, the potentiation of the interaction of growth factors with their target cells, and the acceleration of angiogenesis.^59,60^ A high scaffold swelling ratio supports this, and the significantly greater swelling ratio seen in both layers of the EDAC cross-linked 0.5% bilayered scaffold demonstrates the potential to retain exudate and moisture in the wound microenvironment. It is important to note that the ultimate purpose of the dermal CG scaffold layer is to provide an ECM-based template to promote infiltration of cells into the wound environment and support new tissue formation before reabsorption into the body, which swelling of the scaffold can help facilitate. However, before new tissue is formed, an appropriate resistance to degradation is crucial. In the case of chronic wounds such as DFUs, elevated levels of collagenases such as matrix metalloprotease (MMP)-1 and MMP-8 are found.^61^ While all bilayered scaffolds were stable under hydrolytic conditions (PBS), the EDAC cross-linked scaffolds showed no weight loss under enzymatic conditions over the course of the study. The chemical cross-linking of the collagen fibers allowed the scaffold to resist degradation and maintain its structural stability in an enzymatic environment. The resistance of the EDAC cross-linked scaffolds to degradation over this time period is crucial as it needs to maintain structural integrity to allow for cell infiltration into the wound environment to support tissue growth, before it is replaced by new tissue.^62^

Having confirmed that the epidermal CCh film maintained a continuous top surface for its barrier function, biological characterization of its antimicrobial activity followed. With *S. aureus* being the most common bacterial isolate found in diabetic foot infections,^36^ the ability of the epidermal CCh films to inhibit its growth and infiltration into the wound environment is essential for the prevention of infection during wound healing. All CCh film compositions, regardless of cross-linking treatment, successfully and significantly inhibited the growth of this bacteria. Having determined that the 0.5% bilayered scaffold was the most structurally stable, we showed that the epidermal CCh layer of this bilayered scaffold also prevented the infiltration of *S. aureus* into the dermal CG layer of the scaffold. Clinically, this is important as it would prevent bacterial colonization of the wound. This gives this novel bilayered scaffold a potential advantage over other products, such as the Integra Dermal Regeneration Template, which has a silicone film layer that must be removed as it cannot naturally degrade into the body and is replaced with an epidermal autograft^63^ to allow for full healing of the wound. The CCh film layer consists of biodegradable biomaterials, and it has been shown that chitosan films promote advanced healing, cell proliferation, and re-epithelialization of wounds *in vivo*.^64^ Avoiding the need for further intervention would reduce cost and risk of infection and enhance patient welfare. While the assessment of the antimicrobial activity of the CCh film was limited to *S. aureus*, ¸this is the most common bacterial isolate found in the diabetic wound environment,^36^ and chitosan’s ability to inhibit the growth of many bacteria, both Gram-positive and -negative, is well established.^65,66^ In fact, incorporation of chitosan into biomaterials has endowed antimicrobial activity against various bacteria including *S. aureus*, *Escherichia coli*, and *Pseudomonas aeruginosa*.^67,68^

With the antimicrobial activity of the film confirmed, as well as its function as a barrier to potential infiltrating bacteria, the epidermal CCh film was tested as a surface that supports re-epithelialization of the wound during healing. To create a new barrier between the wound and the environment, keratinocytes located at the wound edge proliferate across newly formed tissue once activated.^69^ It was demonstrated that the epidermal CCh films, regardless of composition or cross-linking treatment, supported the proliferation of keratinocytes with no signs of cytotoxicity. The film supported keratinocyte attachment and proliferation and, when compared to a collagen-only control, demonstrated that incorporation of chitosan into the epidermal film layer of the bilayered scaffold not only provided antimicrobial properties but also did not impact keratinocyte viability. Previous studies have shown that chitosan-incorporated wound dressings promote accelerated re-epithelialization of wounds,^70,71^ giving further evidence to suggest that the epidermal CCh film of the bilayered scaffold provides a suitable surface for re-epithelialization of the wound.

Having determined that the EDAC cross-linked 0.5% bilayered scaffold had the highest structural stability and inhibited the growth and infiltration of bacteria into the wound environment, as well as supporting the proliferation of epidermal cells for re-epithelialization, we then showed that the dermal CG scaffold layer supported the proliferation of vascular cells and promotion of angiogenesis. Accounting for 80% of the dermis,^72^ a type I collagen-based scaffold provides an ideal biomimetic ECM that cells recognize and will proliferate and migrate within. When assessing the ability of the dermal CG layer of the scaffold to support the proliferation of vascular cells, hiPSCs were used, which offer advantages over primary cells as hiPSCs share the same genetic background, providing more reproducible studies. While cell growth was seen in the NXL dermal CG scaffold layer, the elongated vascular cell morphology and increased proliferation observed in the cross-linked scaffolds after 7 days are indicative of the advantages conferred by the improved stiffness of the dermal CG scaffold layer. The increased compressive modulus of the DHT and EDAC cross-linked scaffolds allows them to maintain their pore structure, allowing for the continued proliferation and infiltration of the vascular cells into the dermal scaffold.^22^ This increase in proliferation, coupled with the possible denaturation of collagen due to local unfolding of the collagen triple helix ultrastructure following DHT cross-linking,^73^ encouraged the elongation and early organization of the iECs and iSCs into vascular tubelike structures observed only in the EDAC cross-linked CG scaffolds. This demonstrates the potential of the EDAC cross-linked dermal CG scaffold to support infiltration and migration of vascular cells—critical initial steps in angiogenesis and the formation of vascular networks. Taken together, these *in vitro* results indicate that the 0.5% EDAC cross-linked bilayered scaffold shows great potential as a treatment for diabetic wound healing by preventing wound infection and supporting angiogenesis. However, given the complex nature of diabetic wounds, it is necessary to assess the scaffold in a preclinical model in order to fully establish its potential. Future studies will assess the therapeutic efficacy of the scaffold *in vivo* in a diabetic wound model.

## 5 Conclusions

In this study, we report the development and *in vitro* assessment of a biomimetic, antimicrobial scaffold for the treatment of complex wounds such as DFUs. This bilayered scaffold has intrinsic properties which prevent infection, support re-epithelialization in the epidermal CCh film layer, and promote angiogenesis in the dermal CG scaffold layer. Biophysical and biological characterization showed that the 0.5% EDAC cross-linked bilayered scaffold had the highest structural stability, with similar mechanical properties to products on the market and a similar structure to native skin, and successfully inhibited the growth and infiltration of *S. aureus*. This scaffold also demonstrated the ability to support the proliferation of key cell types involved in vascularization, with early signs of organization of these cells into vascular structures, showing great promise for the promotion of angiogenesis.

## Supporting Information Available

Values from pore-size analysis using toluidine blue staining (Figure S1) (PDF)

## Abbreviations

DFU(s): diabetic foot ulcer(s)
CCh: collagen/chitosan
CG: collagen–glycosaminoglycan
EDAC: 1-ethyl-3-(3-(dimethylamino)propyl)carbodiimide
iPSC(s): induced pluripotent stem cell(s)
iEC(s): induced pluripotent stem cell derived endothelial cell(s)
iSC(s): induced pluripotent stem cell derived stromal cell(s)
ECM: extracellular matrix
C6S: chondroitin-6-sulfate
DHT: dehydrothermal
HaCaT: human keratinocyte
DMEM: Dulbecco’s Modified Eagle Medium
FBS: fetal bovine serum
EGM2: endothelial growth medium-2
SEM: scanning electron microscopy
