# Supplementary material for: A Biomimetic, Bilayered Antimicrobial Collagen-Based Scaffold for Enhanced Healing of Complex Wound Conditions
Source: ACS Appl Mater Interfaces. 2023 Mar 31;15(14):17444–58. doi: 10.1021/acsami.2c18837 (PMC10103052; doi:10.1021/acsami.2c18837)
Supplement: Supplementary file 1 — am2c18837_si_001.pdf [file am2c18837_si_001.pdf]

Supporting Information: A Biomimetic, Bi-Layered Antimicrobial Collagen-based Scaffold for Enhanced Healing of Complex Wound Conditions.

Matthew McGrath[1,2], *Karolina Zimkowska*[1,3], *Katelyn J. Genoud*[1,2,4], *Jack Maughan*[1,2,5,6], *Javier Gutierrez Gonzalez*[1,2,7], *Shane Browne*[1], *Fergal J. O'Brien*[1,2,4]*

1. Tissue Engineering Research Group, Department of Anatomy & Regenerative Medicine, Royal College of Surgeons in Ireland (RCSI), 123 St. Stephen's Green, Dublin, D02 YN77, Ireland

2. Advanced Materials and BioEngineering Research (AMBER) Centre, RCSI and TCD, Dublin, D02 PN40, Ireland

3. Regenerative Medicine Institute, University of Galway, Galway, H91 TK33, Ireland

4. Trinity Centre for Biomedical Engineering, Trinity College Dublin, Dublin 2, D02 PN40, Ireland

5. School of Physics, University of Dublin, Trinity College Dublin, Dublin, D02 PN40, Ireland

6. Centre for Research on Adaptive Nanostructures and Nanodevices (CRANN), Trinity College Dublin, Dublin 2, D02 W085, Ireland

7. School of Chemistry, University of Dublin, Trinity College Dublin, Dublin 2, D02 W085, Ireland

*Corresponding Author: Tel: +353-1-4022149, email: fjobrien@rcsi.ie

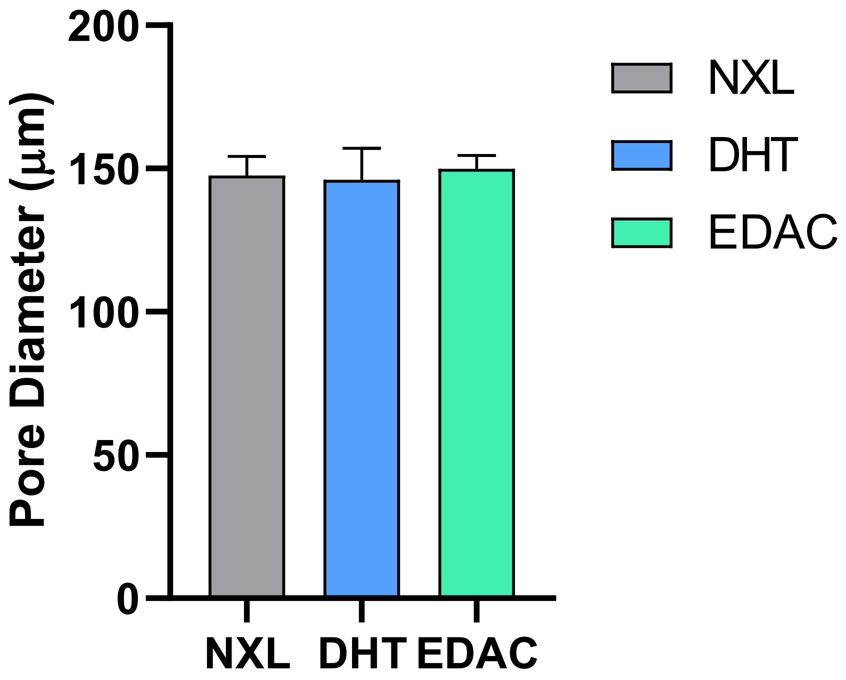

Figure S1. Crosslinking of the bi-layered scaffold did not alter pore size in the dermal CG scaffold layer. Analysis of the pore size was carried out following JB-4 embedding and toluidine blue staining. Pore sizes were determined to be 148 ± 7 μm, 146 ± 11 μm, and 150 ±

5 μm in the NXL, DHT and EDAC CG scaffolds respectively. Data shown are mean ± SD (one-way ANOVA; Tukey's post-hoc test) (N = 3).
